# Ocean acidification with (de)eutrophication will alter future phytoplankton growth and succession

**DOI:** 10.1098/rspb.2014.2604

**Published:** 2015-04-07

**Authors:** Kevin J. Flynn, Darren R. Clark, Aditee Mitra, Heiner Fabian, Per J. Hansen, Patricia M. Glibert, Glen L. Wheeler, Diane K. Stoecker, Jerry C. Blackford, Colin Brownlee

**Affiliations:** 1 Centre for Sustainable Aquatic Research, Swansea University, Swansea SA2 8PP, UK; 2 Plymouth Marine Laboratory, Prospect Place, Plymouth PL1 3DH, UK; 3 Marine Biological Section, University of Copenhagen, Strandpromenaden 5, 3000 Helsingør, Denmark; 4 University of Maryland Center for Environmental Science, Horn Point Laboratory, PO Box 775, Cambridge, MD 21613, USA; 5 Marine Biological Association, Citadel Hill, Plymouth PL1 2PB, UK

**Keywords:** ocean acidification, eutrophication, primary production, plankton succession, food security

## Abstract

Human activity causes ocean acidification (OA) though the dissolution of anthropogenically generated CO_2_ into seawater, and eutrophication through the addition of inorganic nutrients. Eutrophication increases the phytoplankton biomass that can be supported during a bloom, and the resultant uptake of dissolved inorganic carbon during photosynthesis increases water-column pH (bloom-induced basification). This increased pH can adversely affect plankton growth. With OA, basification commences at a lower pH. Using experimental analyses of the growth of three contrasting phytoplankton under different pH scenarios, coupled with mathematical models describing growth and death as functions of pH and nutrient status, we show how different conditions of pH modify the scope for competitive interactions between phytoplankton species. We then use the models previously configured against experimental data to explore how the commencement of bloom-induced basification at lower pH with OA, and operating against a background of changing patterns in nutrient loads, may modify phytoplankton growth and competition. We conclude that OA and changed nutrient supply into shelf seas with eutrophication or de-eutrophication (the latter owing to pollution control) has clear scope to alter phytoplankton succession, thus affecting future trophic dynamics and impacting both biogeochemical cycling and fisheries.

## 1. Introduction

Effects of ocean acidification (OA), and specifically the impacts of higher *p*CO_2_ and a lower pH (higher [H^+^]) on marine life are subjects of much research. Taken together with other climate change events, notably changes in temperature and water-column stability, OA has the potential for various impacts upon marine plankton communities and production [1–9]. The removal of CO_2_ by primary production leads to seawater basification (increase in pH), and the tendency towards this event is enhanced by increased nutrient availability. Indeed, in coastal waters subjected to eutrophication by addition of inorganic nutrients that support large algal blooms, the increase in pH can be highly significant and can override any signal from OA [10]. On the contrary, when organic eutrophication promotes increased system respiration (net addition of CO_2_), then acidification is increased [11]. Events can also run in series, with inorganic eutrophication promoting first primary production and then respiration during decay of the bloom biomass. Either way, the system pH displays variations that may affect ecology, and these events will be affected with OA by commencement at a lower pH. Such transients are of most consequence in the highly dynamic coastal zones which contain elevated nutrient loadings in comparison with the relatively stable and low nutrient oligotrophic oceans.

Significant basification during bloom development directly influences phytoplankton species growth rates and succession [12–14], selecting species most capable of growth as pH increases [13–16]. Further, the major grazers of phytoplankton, the microzooplankton, can be sensitive to elevated pH [17], just as copepods can show sensitivity to lower pH [8]. Such selective pressures affect the extent of biomass production and also its fate. These pH-sensitive events in future oceans will be affected by the long-term decrease in ocean pH with OA overlain by short-term changes in seawater pH such as basification during phytoplankton blooms. Because of climate change (including changes in terrestrial runoff), human population growth and allied changes in land use, enhanced nutrient release from the land is also expected over the coming decades. There are expected to be particularly severe regional impacts affecting coastal marine ecosystems [18,19], though some areas may see reversal of eutrophication under policies to decrease pollution. Any changes in nutrient loading that affect primary production will also affect the degree of basification.

The implications of the potentially synergistic effects of (de)eutrophication and OA, inducing modifications to phytoplankton growth and species succession, have not previously been explored. With OA, bloom-induced basification will commence at a lower pH. This will fundamentally shift the conditions for competitive interactions between bloom-forming species over a different range of H^+^ concentrations, with potentially unanticipated impacts on predator–prey dynamics through to higher trophic levels. Any significant changes to plankton growth in estuarine, coastal and shelf sea systems will have profound implications owing to the substantial contribution these systems make to global productivity [18,19] and marine ecosystem services [20].

This paper explores how these contrasting drivers on seawater pH (pH declining with OA versus basification with inorganic nutrient eutrophication) may affect growth and interactions of different types of phytoplankton. We show, using a combination of experiments and models, that future changes in ocean carbonate chemistry, coupled with changes in the availability of nutrients, will have an important influence on competitive interactions between phytoplankton taxa during bloom development.

## 2. Material and methods

A brief overview is provided here; detailed methods are described in [21] and in the electronic supplementary material. Three taxonomically contrasting phytoplankton, a weakly calcifying strain of the non-motile prymnesiophyte (*Emiliania huxleyi*), a motile cryptophyte (*Rhodomonas* sp.), and a silicifying diatom (*Thalassiosira weissflogii*), were grown in nitrogen-limiting cultures. Initial pH treatments were set as typical of present day seawater (extant: pH 8.2), future OA (acidic: pH 7.6) or within dense blooms (basic: pH 8.8). pH was then allowed to drift with phytoplankton growth with no CO_2_ entry to counter dissolved inorganic carbon (DIC) removal through primary production. (In the oceans, gas exchange to equilibrium is very slow, especially into a mixing depth of many metres.) These pH treatments are henceforth termed ED (start at extant pH with drift), AD (start at acidic pH with drift) and BD (start at basic pH with drift). In addition, comparative treatments were run with pH held fixed at the initial values by addition of HCl or NaOH; these conditions are termed EF (extant pH fixed), AF (acidic pH fixed) and BF (basic pH fixed). Data from experiments (nutrients, biomass C, N and P) were used to configure multi-nutrient, photoacclimative, variable stoichiometry models of phytoplankton growth dynamics, which were coupled to a carbonate chemistry submodel, as described in [22]. These models were then run under different scenarios of nutrient and *p*CO_2_ (OA), and of mixing depth, to simulate growth of single or multi-species phytoplankton communities.

## 3. Results

**Figure 1.**
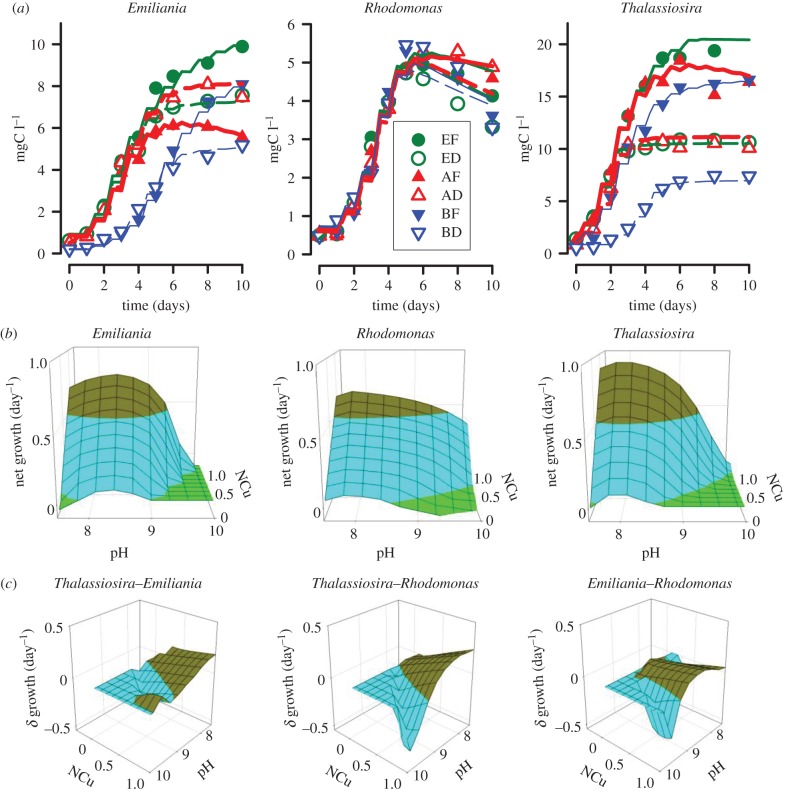
Experimental data and model outputs. (*a*) Experimental data (symbols) for the prymnesiophyte *Emiliania huxleyi*, the cryptophyte *Rhodomonas* sp*.* and the diatom *Thalassiosira weissflogii* grown under conditions of pH that were extant fixed at pH 8.2 (EF), extant drifting from pH 8.2 (ED), acidic fixed at pH 7.6 (AF), acidic drifting from pH 7.6 (AD), basic fixed at pH 8.8 (BF) or basic drifting from pH 8.8 (BD). Experimental data are averages from duplicate experiments, with the range of those values typically within the symbol size. Lines are model fits to the data. (*b*) Emergent relationships between growth rate, pH and nutrient status (NCu; where 0 is nutrient-starved and 1 is replete). Measured pH ranged between 7.5 and 10; simulation outputs are shown within these values. Darkest zones (brown in online colour plot) indicate zones with growth rates >0.5 day^−1^. (*c*) Differences in net growth potential (*δ* growth) between pairs of algae, with pH and nutrient status. In each plot, the light zones (blue in online colour plot) indicate where the second named species would outgrow the first named species; a value of *δ* growth = zero indicates where neither species exhibited positive net growth. (Online version in colour.)

In comparison with growth in the extant drift (ED) pH systems, growth of *Thalassiosira* and *Emiliania* was almost halved in the basic drift (BD) systems, and was similar or slightly enhanced in the acidic drift (AD) systems (figure 1*a*). There was little difference between *Rhodomonas* grown in drift systems of different initial pH, at least during the nutrient-replete phase. Typically, growth of these single species cultures was greater under fixed-pH rather than under drift-pH conditions (figure 1*a*), with DIC draw-down continuing until concentrations of substrates for photosynthesis (H_2_CO_3_ and HCO_3_^−^) were very low (electronic supplementary material, figures S1, S3–S5). Cessation of growth at high pH in drift systems was thus not a simple consequence of the exhaustion of DIC. Only for *Emiliania* was growth under AF pH conditions poorer than under AD pH conditions (figure 1*a*). Within multi-species cultures, *Emiliania* biomass declined following nutrient exhaustion; this could not be explained simply through reference to the results from single species cultures (figure 1 and the electronic supplementary material, figure S1 versus S6), but appears to be related to the extent of growth of total biomass (which was greater in fixed-pH systems), perhaps associated with a lack of DIC and/or an allelopathic (or other cell–cell) interaction.

Fits of the model to experimental data (figures 1*a* and the electronic supplementary material, figures S1 and S2) were achieved by inclusion of functions relating both phytoplankton growth and death to pH and cellular nutrient status (electronic supplementary material, table S1). The optimal pH, and form of the interaction between pH with nutrient stress, differed between species (figure 1*b*). When emergent relationships were compared for pairs of species (figure 1*c*), potential windows of opportunity for each competing species became apparent. Thus, *Emiliania* growth is favoured under extant pH, with more acidic and basic conditions being unfavourable. *Rhodomonas*, which had the lowest maximum growth rate, has its highest competitive scope at elevated pH but only when nutrient-replete; these are conditions likely associated with eutrophic areas during blooms.

Dynamic sensitivity analyses, conducted on simulations for the multi-species cultures under the fixed- and drift-pH regimes (electronic supplementary material, figures S7–S9), indicated that phytoplankton succession is most predictable (i.e. least variable) under extant pH, and least predictable under the acidic (OA) scenarios. This reflects the form of the relationship between pH–nutrient status–growth/death for each species (figure 1*b*), tracking consumption of DIC and nutrients during growth of the total algal community. Variability in response was further enhanced by the decreased buffering capacity of seawater at lower pH [22], which affects growth under the acidic scenarios. Even within a narrow pH range, the additional impact of nutrient stress can be significant (figure 1*c*); the prymnesiophyte *Emiliania* showed greater sensitivity and relatively lower competitive advantage in the acidic fixed-pH (AF) simulations.

**Figure 2.**
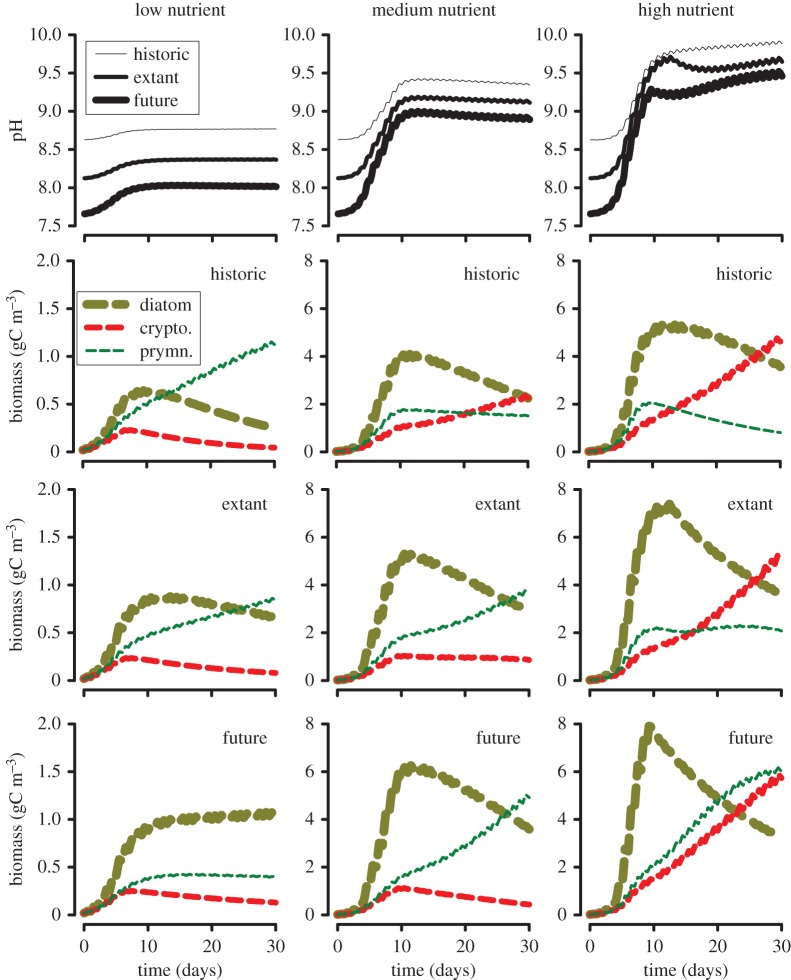
Simulations of competitive growth between three phytoplankton types under different *p*CO_2_ scenarios and physico-chemical characteristics. The plankton type models conform to the cryptophyte (crypto.), diatom (diatom) and prymnesiophyte (prymn.) types, as configured against experimental data (figure 1*a*). Initial algal-C biomass values were the same for each type; biomass has units of gC m^−3^. Scenarios conformed to historic (preindustrial, *p*CO_2_ 280 ppm), extant (*p*CO_2_ 390 ppm) and future (prediction for 2100, *p*CO_2_ 1000 ppm) conditions. Nutrients (N, P, Si) were supplied at Redfield ratios; the low nutrient regime contained 5 µmol N l^−1^ with mixing depth of 40 m; the medium nutrient regime contained 40 µmol N l^−1^ with mixing depth of 10 m; the high nutrient regime contained 200 µmol N l^−1^ but with only 40 µmol Si l^−1^ with mixing depth of 5 m. In all instances wind speed was set at 10 m s^−1^, maximum day time surface irradiance at 2000 µmol photons m^−2^ s^−1^ in a 12 : 12 h light : dark cycle, mixing rate between upper and lower layers of 0.05 day^−1^, temperature of 16°C and salinity of 35. (Online version in colour.)

Simulations were also made under different conditions of gas exchange equilibration to historic, extant or future atmospheric *p*CO_2_, low medium and high nutrient loadings, and mixing depths (figure 2). These plots should be viewed as being indicative of the potential for different successions, and not necessarily that one or other plankton group (diatom versus cryptophyte versus prymnesiophyte) will dominate in nature under a given set of conditions. Simulated future scenarios generally gave rise to faster and more extensive growth (figure 2), and more extreme elemental stoichiometry (electronic supplementary material, figure S10) when light or Si was not limiting. Simulated bloom composition varied between *p*CO_2_ scenarios, because the deleterious conditions at elevated pH were encountered later during bloom development under future scenarios (the starting pH being lower with OA).

## 4. Discussion

Our study shows species variability within the pH–growth relationship and how this is affected by nutrient stress (figure 1*b*). Predicted under different OA and (de)eutrophication scenarios (figure 2), the implications of our results for shifting plankton successions in shelf seas are numerous and far reaching. Over time, some waters (e.g. North Sea) will have seen changes from conditions akin to historic *p*CO_2_ with mesotrophy, to extant *p*CO_2_ with eutrophy, to future *p*CO_2_ with mesotrophy, with corresponding scope for significant changes in plankton dominance (figure 2). More broadly, with greater inorganic eutrophy (an increasingly likely event [18,19,23]) coupled with elevated *p*CO_2_, there is potential for an increase in total bloom size and/or of the rate of growth in coastal and shelf seas. The nutrient balance in shallow waters is also affected by basification during blooms leading to the release of bound nutrients [23], and decreased coupling of nitrification–denitrification [24]. With OA, initiation of all these events will occur at a lower average pH, but additional differences in bloom extent and composition as a consequence of other impacts, including temperature changes and pollution control measures may be expected [10,25].

The responses of our coupled experimental–model systems, with respect to growth dynamics and C : N : P, are consistent with empirical studies [1,2,9,26], providing confidence in the behaviour of the models. However, dominance of different taxa in nature will also be affected by other factors, including responses of the zooplankton. Blooms develop only in the absence of effective grazing pressure [27]. The activity of grazers will have several points of interaction. More extreme stoichiometry (electronic supplementary material, figure S10) may be detrimental to grazers [27,28], and also promote toxicity in some phototrophic species [29]. High pH may decrease net growth of grazer populations [17] and hence decrease feeding on phytoplankton, whereas low pH has been seen to affect vertical migration of flagellates [30] and copepod growth [8]. Nonetheless, all these interactions are ultimately secondary to the auto-ecological responses of individual primary producers to nutrients, pH and light.

The fate of this changed primary production, depending on local conditions and bloom composition, when coupled with impacts on grazers, will likely be associated with increased frequency of deleterious events such as harmful and ecosystem-disruptive algal blooms [31,32], and increases in hypoxic and anoxic zones, affecting fisheries [33] and thence food security. For blooms of calcifying phototrophs, and any plankton growing within these blooms, the situation is complicated further as calcification with primary production mitigates pH increases [22]. If coccolithophore growth is adversely affected by lower pH (as seen here), then there is opportunity for enhanced variability in their bloom development; this will depend on how preceding non-coccolithophorid primary production conditions affect the water column (figures 1*b*,*c* and 2).

Our work shows variation in the sensitivity of phytoplankton commencing growth under different initial conditions to changes in pH (figure 2 and the electronic supplementary material, figures S7–S9). While there has always been variability in seawater pH, with OA, the variability in [H^+^] will be greater, starting from a less buffered state at a higher [H^+^] [22,34]. Studies on OA, therefore, need to consider the dynamic interplay between primary and secondary producers grown under variable pH, and their physiological mechanisms to cope with these dynamics. While one may expect organisms growing with OA to adapt to lower pH [35], it is uncertain whether such adaptations would then adversely affect scope for growth over the broader pH range with bloom-induced basification. Further, as nutrient stress impacts negatively on the ability to cope with pH-induced stress (figure 1*b*), the potential for mixotrophs, with their ability to derive nutrients from different sources [36,37], to be favoured under OA scenarios also warrants research. Taken together, we have every reason to expect a more variable and less predictable future for primary production in coastal and shelf seas.

## Supplementary Material

Detailed Methods and Additional Figues

## Data accessibility

Raw experimental data used in this work are available from the corresponding author; these are graphically displayed in this work and its allied electronic supplementary material. Model code is available in, and is described in, the electronic supplementary material; code may also be obtained in the form used for this work (i.e. as a Powersim Constructor file) from the corresponding author.
