# Supplementary material for: Ocean acidification with (de)eutrophication will alter future phytoplankton growth and succession
Source: Proc Biol Sci. 2015 Apr 7;282(1804):20142604. doi: 10.1098/rspb.2014.2604 (PMC4375859; doi:10.1098/rspb.2014.2604)
Supplement: Detailed Methods and Additional Figues [file rspb20142604supp1.pdf]

Supplementary Materials

Materials and Methods

Experimental

Cultivation of algae - Axenic cultures of the prymnesiophyte *Emiliania huxleyi* (Lohm.) Hay and Mohler (strain L, for strain details see [38]; equivalent cell diameter ca. 4 µm), the cryptophyte *Rhodomonas* sp (CCMP 775; equivalent cell diameter ca. 8 µm), and the diatom *Thalassiosira weissflogii* (Hustedt) Hasle et Heimdal (CCMP 1010; equivalent cell diameter ca. 15 µm), were grown in batch culture over 10 days in a constant temperature room at 16(±1) °C. Illumination was provided by 4 cool white fluorescent tubes (Philips Master TL-D/70W/840/6200 lm) at a photon flux density of 240 µmol photon·m^{-2}·s^{1} (PFD; Biospherical Instruments QSL 100 meter with a 4π detector, San Diego, CA, USA; waveband 400–700 nm), as measured at the centre of water-filled experimental containers, in a 18:6 h light:dark cycle.

The initial nutrient concentrations, with nitrate as the N source, were intended to result in N-limiting conditions. All treatments were duplicated. Cylindrical experimental flasks (10 L) were filled with 9 L of culture, of which 3 L were removed for experimental sampling over the time course of the experiment. Culture media was based on artificial seawater (ASW; [39]) with a bicarbonate concentration of 2.0 mmol·L^{-1} and no organic buffer. Modifications and protocols were used [21, 40, 41], which take into consideration the improved purity of reagent grade chemicals used for ASW media over those of the original formulation. De-ionized and purified water (ELGA Purelab ultra, VWS UK Ltd., High Wycombe, UK) and chemicals of analytical grade were used for media preparation. Following this protocol the pH of complete ASW was ca. 8.2.

Under aseptic conditions ASW was filter-sterilized by pumping it into autoclaved experimental flasks through autoclaved 47 mm diameter, 0.2µm filters (Durapore, Merck Millipore, Billerica, MA, USA). Enrichment Solutions (ES) for vitamins, iron, and trace metals of 10% of the original ASW [41] were added to decrease the amount of EDTA and hence the amount of non-utilizable dissolved organic carbon and nitrogen (DOC and DON) in the medium. Due to the relatively low macro-nutrient concentrations (compared to alternative growth media), ES additions were inferred to be non-limiting. Nitrate was supplied as the limiting nutrient (25 µmol·L^{-1} for the non-diatoms, 50 µmol·L^{-1} for the diatom). Phosphate was supplied at non-limiting concentration (20 µmol·L^{-1}), plus for the diatom cultures silicate was also supplied at non-limiting concentration (134 µmol·L^{-1}).

Axenic stock cultures were used to start experiments, and precautions taken to minimise the risk of any subsequent contamination in experimental flasks. No significant contamination (as judged by microscopy) was noted during experiments. Two- point calibrated pH probes (pH 7.01 and 10.01; HI 1131b, Hanna Instruments, Woonsocket, RI, USA) were surface sterilized with 2 mol·L^{-1} HCl for 15 min and rinsed with sterile pure water before being inserted into experimental vessels under aseptic conditions. Experimental vessels were sealed gas-tight with sterilized silicon bungs through which passed the pH probe lead, a sampling tube, two further tubes which facilitated pH correction, and a tube for the entry of N_2 gas to replace the liquid volume removed in sampling.

A bespoke software based pH monitoring and control unit was constructed at Plymouth Marine Laboratory (U.K.) for use in these experiments. Using this, cultures were independently monitored and, as required, the pH controlled. The unit was operated in 2 modes; a "fixed" mode where pH was maintained at a set value throughout the experiment, and a "drift" mode in which pH was monitored as it varied from a pre-determined starting point due to phytoplankton growth (increasing with net C-fixation, and decreasing with net respiration overnight). The values at which pH was either fixed, or from which it drifted, were pH 8.2 representing extant conditions and pH 7.6 ("acidic" treatments) representing an acidified ocean. In addition, a series of experiments commencing at an elevated pH (8.8; "basic" treatments) were also run.

The pH values were measured using a bench top pH meter (HI 931400, Hanna Instruments, Woonsocket, RI, USA). Both temperature, measured in tap-water filled vessels placed adjacent to experimental flasks to avoid the potentially toxic effects of stainless steel probes (HI 7669/2W, Hanna Instruments, Woonsocket, RI, USA), and culture pH were measured at a time step of 70 seconds. For cultures at fixed pH, a tolerance of 0.05 pH units was assigned and 5 consecutive measurements beyond this tolerance resulted in pH correction; metering pumps added either 1 mL of HCl or NaOH to individual cultures. During the growth cycle in these closed system cultures, dissolved inorganic carbon (DIC) concentration and buffering capacity decreased. For cultures grown at fixed pH the concentration of acid/base added to cultures was decreased as culture age increased in order to minimize the amplitude of pH change during the automated correction procedure. An initial HCl/NaOH concentration of 1.2/0.25 µmol·L^{-1} was used for the acclimation stage and first experimental day, decreasing to 0.12/0.125 µmol·L^{-1} at day 2 and 0.05/0.0625 µmol·L^{-1} at day 4 respectively. A log of temperature, pH and acid/base addition for each experimental vessel was recorded by the pH control unit. Changes in total alkalinity (TA) were calculated from changes in nutrient (including DIC) and pH by reference to methods used previously [22]; see model description, below.

Inoculated experimental containers were placed on magnetic stirrers within a constant temperature room, connected to the pH control unit, and kept for 14 to 16 hours for temperature and light acclimatization. During this stage pH was maintained at a constant level of 7.6, 8.2 or 8.8 as required. After acclimatization, pH control was switched to “drifting” or “fixed” modes to initiate experiments (t0).

Sample collection - Samples were collected from vessels on a daily basis for days 0-6, and then at days 8 and 10. Samples were drawn from a PTFE tube that was permanently submerged in culture media. The first 10 mL of drawn media was discarded, and the subsequent 250-450 mL retained. N_2 gas was introduced to the atmosphere above cultures to replace the volume removed by sampling. Sample volume was stored in the dark at 4 °C and sub-sampled for a range of procedures which were completed within 4 hours.

Cell numbers and biovolume - Cell numbers and biovolume of sub-samples were measured with a FlowCam (single species experiments with *Rhodomonas* sp and *T. weissflogii*; Fluid Imaging Technologies, Yarmouth, ME, USA) equipped with 90 µm field of view flow cell and a x10 objective, and/or with a Coulter Counter (*E. huxleyi* and triple species experiments; Multiplier 4, Beckman Coulter GmBH, Krefeld, Germany). For the FlowCam, image data processing software “Visual Spreadsheet” was used (version 2.1.20 beta, FlowCam and software: Fluid Imaging Technologies, Yarmouth, ME, USA). Analyses of particles of the size range of 5 to 30 µm were carried out with a picture rate of 6 s^{-1} in auto-image mode. Images in which algal cells were absent were excluded following visual examination of images. Typically, 500 particles were counted although in low density cultures measurements were stopped after 4 minutes to avoid sedimentation effects. Biovolume was computed using the ABD FlowCam algorithm, which equates to a volume estimated from equivalent spherical diameter. Data represent the median of at least 3 replicate measurements.

Analysis of inorganic and organic nutrients - Under clean conditions (within a filtered air stream in a laminar flow cabinet), sub-samples of culture were gravity filtered through pre-combusted (550 °C, 20 min) glass fibre filters with a nominal pore size of 1 µm (Type A/E, 13 mm, Pall Corporation, Ann Arbor, MI, USA). Filters, which retained cells, were stored frozen for subsequent elemental and compositional analyses.

A segmented flow auto-analyzer (model AA3, Seal Analytical Ltd., Fareham, UK), consisting of a random access XY-2 auto-sampler, a high precision pump, chemistry module trays and a dual

beam high resolution digital colorimeter operating within the range 340-900 nm, was used for inorganic analyses. Nitrate and nitrite were determined by the cadmium reduction method and sudan-1 synthesis, measured at 550 nm. The determination of silicate was based on the reduction of silico-molybdate in acidic solution to molybdenum blue by ascorbic acid and the coloured complex was measured at 820 nm. The Berthelot reaction was used for the determination of ammonium with indophenol measured at 660 nm. The analysis of inorganic phosphorous [42] produced a blue phosphor-molybdenum complex measured at 880 nm. The determination of DIC was based [43] on inverse chemistry with the use of buffered phenolphthalein indicator and was measured at 550 nm.

Analysis of particulate C, N and P - Particulate C and N content were analyzed against isoleucine standards using an elemental analyzer and mass spectrometer (ANCA PDZ-Europa and 20-20 IRMS PDZ-Europa, SerCon Ltd Crewe, UK) combined with Callisto 530 software (SerCon Ltd Crewe, U.K.). Both samples and standards were wrapped in tin (Sn) foil. Helium gas served as system carrier and auto-sampler purge. The combustion column, which was packed with quartz wool/chromium oxide/silver wool, operated at 1000 °C, with sample combustion taking place in the presence of an O_2 pulse to improve combustion efficiency. Combustion gases were reduced in a reduction column composed of silica wool/copper wire at 600 °C. Sample derived CO_2 and N_2 gases were separated by a gas chromatography column at 70 °C and analyzed by mass spectrometry.

Particulate P content was derived by alkaline persulphate oxidation followed by analysis of the solubilized phosphate. Algal samples retained on glass fibre filters were heated for 20 minutes at 121 °C (autoclave) in sealed glass ampoules containing 2 mL of a solution containing potassium peroxodisulphate (50 $g \cdot L^{-1}$), boric acid (30 $g \cdot L^{-1}$) and sodium hydroxide (15 $g \cdot L^{-1}$). After shaking, 1.5 mL of the supernatant was pipetted into micro-centrifuge tubes, glass fibre remains were removed by centrifugation (20 000 x G for 5 minutes) and 1 mL of supernatant was stored frozen (-20 °C) until analysis. Inorganic P content was measured in samples diluted using purified water (ELGA Purelab ultra, VWS UK Ltd., High Wycombe, U.K.) and analyzed as phosphate in the segmented flow auto-analyzer (as described for nutrient phosphate, above).

Commentary on experiment design

The experimental design deployed was used in an attempt to achieve a mass balance for carbon across all forms of carbon in the system (see [21]). To this end, the systems were sealed to prevent ingress of CO_2, and pH was controlled by acid/base additions rather than through changes in pCO_2. It is noteworthy that in natural systems the equilibration of the atmosphere with the surface mixed layer

(which may comprise several 10s of meters depth), is a slow process, of the order of months. Preventing gas exchange in the experiments over a period of 10d does not thus induce an unnatural scenario.

The method used for control of pH (addition of acid/base) introduces the potential for significant changes in alkalinity, and is not the favoured approach for studying ocean acidification [44,45]. However, for the processes explored in this work attempting to rigidly maintain a constant pH (by any means) is artificial because the events in question centre around the fact that seawater pH is not constant but changes with biological activity.

The primary experimental data used for this work, for the tuning (calibration) of the models, comes from the “drift” systems, which were not subjected to any additions of acid or base other than that required for the initial setting of pH (“extant”, “acidic” or “basic”). The initial chemistry of the “drift” flasks thus differed only slightly with respect to total alkalinity (TA). The fact that the highest rates and extent of growth were seen in fixed pH systems, which underwent very significant changes in TA over the duration of the experiments (figure S1), indicates that TA has no damaging influence on cell physiology, and certainly not one that is discernible against a background of changing DIC and pH in the experimental systems deployed. This subject is discussed further in [21].

Simulations

Modelling was undertaken using the same platform (Powersim Constructor v2.51, Isdalstø, Norway) and source model code as deployed previously [22]. This included a variable stoichiometric acclimative model of phytoplankton physiology, coupled to a carbonate chemistry module. The only additional code added were equations linking growth and death rates to pH and nutrient status; these are provided in Tables S1. The carbonate chemistry component of the model (used also in [22] and in [4]) yields values for the carbonate chemistry system, interrelating pH, TA and DIC, that are very similar to those generated by the CO2sys software [46]. Here, these values (pH, TA, DIC and thence the division of DIC between H_2CO_3, HCO_3^- and CO_3^{--}) were computed during the simulations based on the initial start parameters for pH, TA, DIC, and nutrients, and the fit of the model to the experimental data describing algal growth. The closeness of model fits to the experimental data, and of the emergent pH values from the model versus the logged pH values during experiments (figures S1) provides confidence in the computed values of TA and DIC (figure S1 and figures S3-5).

The model was tuned against experimental data for cell-C and cell N:C quotas derived for each individual organism (see *Experimental*) using the evolutionary tuning method supported by Powersim Solver v2 (Powersim, Isdalstø, Norway). This provided, for each organism, a single set of constants

controlling physiology. Including TA as a parameter during tuning of the models had no effect on the outcome of this modelling analysis.

Dynamic sensitivity analyses – Sensitivity analyses were undertaken using the Powersim Solver v2 platform, running a Latin-hypercube routine with an initial pH set for “extant” (pH 8.2), “acidic” (pH 7.6) or “basic” (pH 8.8) together with a set standard deviation. The standard deviation used for the extant simulations (pH 8.2) was 0.1 pH units. The addition or subtraction of H^+ ions required to achieve this deviation (i.e., pH 8.2 +/- 0.1), generates a different pH shift when commencing at different pH values. This reflects the consequences of the pH scale being a log transform, and also because the buffering capacity of the carbonate system falls rapidly as pH declines below 8 [22,34]. Thus, for a given change in $[H^+]$, the deviation of pH 8.2 +/- 0.1 equates approximately to pH 7.6 +/-0.2 and pH 8.8 +/-0.05.

Simulations of open water pH scenarios - Simulations were run using the phytoplankton models that had been configured against the experimental data, but now within scenarios of a water column with gas exchange at the surface, and exchange of water at the interface of the mixed layer. The basis of these simulations in terms of abiotic factors is the same as used previously [22]. The major differences, other than the lack of simulated predator activity, was that the future pCO_2 was set at 1000ppm (prediction for end of this century [47,48]), rather than 750ppm.

Scenarios conformed to historic (preindustrial, pCO_2 280 ppm), extant (pCO_2 390 ppm), and future (prediction for 2100, pCO_2 1000 ppm) conditions. Nutrients (N, P, Si) were supplied at Redfield ratios; the low nutrient regime contained 5 µmol N L^{-1} with mixing depth of 40 m; the medium nutrient regime contained 40µmol 1 N L-1 with mixing depth of 10 m; the high nutrient regime contained 200 µmol N L^{-1} but with only 40 µmol Si L^{-1} with mixing depth of 5 m. In all instances wind speed was set at 10 m s^{-1}, maximum day time surface irradiance at 2000 µmol photons m^{-2} s^{-1} in a 12:12h light:dark cycle mixing rate between upper and lower layers of 0.05 d^{-1}, temperature of 16 °C and salinity of 35.

References in addition to those in the main text

38. Ietswaart T, Schneider PJ, Prins RA. 1994 Utilisation of organic nitrogen sources by two phytoplankton species and a bacterial isolate in pure and mixed culture. *Appl. Environ. Microbiol.* **60**, 1554–1560.

39. Harrison PJ, Waters RE, Taylor FJR. 1980 A broad spectrum artificial seawater medium for coastal and open ocean phytoplankton. *J. Plankt. Res*. **3**, 331–344.

40. Berges JA, Franklin DJ, Harrison PJ. 2001 Evolution of an artificial seawater medium: Improvements in enriched seawater, artificial water over the last two decades. J. Phycol. **37**, 1138–1145.

41. Andersen RA, Berges JA, Harrison PJ, Watanabe MM. 2005 Appendix A – Recipes for freshwater and seawater media. In: Algal Culturing Techniques (ed Andersen RA), pp. 429–538, Elsevier, Amsterdam.

42. Murphy J, Riley JP. 1962. A modified single solution for the determination of phosphate in natural waters. *Anal. Chim. Acta* **27**, 31–36.

43. Stoll MHC, Bakker KMJ, Nobbe GH, Haese RR. 2001 Continuous-flow analysis of dissolved inorganic carbon content in seawater. *Anal. Chem.* **73**, 4111–4116.

44. Hurd CL, Hepburn CD, Currie KI, Raven JA, Hunter KA. 2009 Testing the effects of ocean acidification on algal metabolism: considerations for experimental designs. *J. Phycol.* **45**, 1236-1251.

45. Riebesell U., Fabry V. J., Hansson L. & Gattuso J.-P. (Eds.), 2010. *Guide to best practices for ocean acidification research and data reporting*. Luxembourg: Publications Office of the European Union.

46. Pierrot D, Lewis E, Wallace DWR (2006). CO2sys MS Excel Program Developed for CO_2 System Calculations. ORNL/CDIAC-105. Carbon Dioxide Information Analysis Center, Oak Ridge National Laboratory, US Department of Energy, Oak Ridge, TN, U.S.A.

47. IPCC (2007) Climate Change. 2007 *The fourth assessment report of the intergovernmental panel on climate change (IPCC)*. Cambridge University Press, Cambridge.

48. van Vuuren DP, Edmonds J, Kainuma M, Riahi K, Thomson A, Hibbard K, Hurtt GC, Kram T, Krey V, Lamarque J-F *et al.* 2011 The representative concentration pathways: an overview. *Clim. Change* **109**, 5–31.

49. Clark DR, Flynn KJ. 2000 The relationship between the dissolved inorganic carbon concentration and growth rate in marine phytoplankton. *Proc. Roy. Soc. Lond. B* **267**, 953-959.

Tables S1 Relationship between growth rate, death, pH and nutrient status

DESCRIPTION OF FUNCTIONS

The growth function describes a bell-shaped curve with optimum, minimum and maximum pH values. The function returns a value between 0 (no growth potential) and 1 (maximum potential). As the nutrient status decreases, so the breadth of pH supportive of growth also decreases (narrows). Thus under optimal conditions of zero nutrient status, growth proceeds at a maximum rate and also over the widest pH range. As the organism becomes stressed, so the ability to withstand pH extremes lessens; the width of the bell narrows and its optimum may change. The growth rate then becomes a function of the incident irradiance and of the nutrient status (both described via the usual functions; see (22) and references therein), and of pH.

The death function describes a default death rate that under optimal nutritional status is relatively flat and low over the pH range. As nutrient stress develops so the death rate even under optimal pH for growth increases, and the rate increases even more so at pH values either side of the optimum value. The function thus resembles a 'U' shape that becomes higher and steeper with increasing nutrient stress.

PARAMETER AND EQUATIONS

The equations and definitions are defined here specifically for the diatom *Thalassiosira* (_diat) description. The tabulated constants provide values for the alternative values for the description of the flagellate *Rhodomonas* (replace _diat with _flag), and microalgae *Emiliania* (replace _diat with _alg).

Equations are given as ASCII text to facilitate their use in models or spreadsheets, and also as traditional formal equations. The equations contain Boolean logic terms, which take the value of 1 if true and 0 otherwise; these may be replaced by IF-THEN-ELSE constructs as required.

Table S1a Descriptions of constants. Note the full name of the constant (in ASCII text format) is the combination of the name prefix plus the organism identifier; e.g. pHopt_diat. In the formal equation syntax, the organism identifier is subscripted; e.g., $pHopt_{diat}$

	CONSTANT	UNIT	DESCRIPTION
GROWTH RATE LINKAGE TO pH	PHopt	pH	optimum pH
	PHmin	pH	minimum pH when nutrient replete
	pHmax	pH	maximum pH when nutrient replete
	NCuH	DL	power on curve link to NCu (small values broaden shape)
	pHK	DL	the lower the value the broader the bell
	pHH	DL	the higher the value the more sigmoidal; balance this with increasing pHK
DEATH RATE LINKAGE TO pH	Dro	d^{-1}	default death rate
	ls	DL	scalar for death impact of low pH
	lNCu	DL	link from nutrient status to death at low pH. 0 gives no link and death is default value
	curv	DL	curve shape for death function
	hs	DL	scalar for death impact of high pH
	hNCu	DL	link from nutrient status to death at high pH. 0 gives no link and death is default value
	Ldd	DL	low default death impact (value 0-1, with 1 being no default)
	Hdd	DL	high default death impact (value 0-1, with 1 being no default)
MAXIMUM GROWTH RATE	Um	d^{-1}	maximum growth rate under continuous illumination

Table S1b Values of constants

	CONSTANT	Diatom (*_diat)	Flagellate (*_flag)	Alga (*_alg)
GROWTH RATE LINKAGE TO pH	pHopt	7.929	7.780	8.41
	pHmin	5.792	6.640	6.00
	pHmax	10.483	10.980	9.868
	NCuH	0.529	0.500	0.506
	pHK	0.428	0.286	0.293
	pHH	3.586	1.690	2.477
DEATH RATE LINKAGE TO pH	Dro	2.13E-02	0.009	0.0005
	Ls	0.749	0.148	0.405
	lNCu	4.098	1.013	2.444
	Curv	2.817	2.159	2.570
	Hs	0.009	0.269	0.013
	hNCu	0.623	5.466	5.323
	Ldd	0.299	0.622	0.263
	Hdd	0.935	0.994	0.565
MAXIMUM GROWTH RATE	Um	1.930	1.389	1.609

Table S1b Descriptions of parameters for growth interactions

PARAMETER	FORMAL NAME	UNIT	DESCRIPTION
**INPUTS**			
pH	pH	pH	pH of medium
NPCu_diat	$NPCU_{diat}$	DL	nutrient status from normalised quota equation (0 = poor; 1 is optimal)
**AUXILIARIES**			
pHminop_diat	$pHminop_{diat}$	pH	operational lower extreme of pH window, as affected by nutrient status
rpHmin_diat	$rpHmin_{diat}$	DL	relative value of pH within the lower pH window
cpHmin_diat	$cpHmin_{diat}$	DL	value of control linked to minimum pH; returns 0 if at or below pHminop; returns 1 if pH>= pH opt
pHmaxop_diat	$pHmaxop_{diat}$	pH	operational upper extreme of pH window, as affected by nutrient status
rpHmax_diat	$rpHmax_{diat}$	DL	relative value of pH within the upper pH window
cpHmax_diat	$cpHmax_{diat}$	DL	value of control linked to maximum pH; returns 0 if at or above pHmaxop; returns 1 if pH<= pH opt
**OUTPUT**			
pHreg_diat	$pHreg_{diat}$	DL	overall pH regulator term (quotient); this is used to modify Umax

Table S1d definitions of parameters for growth interactions

PARAMETER	FORMAL NAME	DEFINITION
**INPUTS**		
pH	pH	input from DIC chemistry
NPCu_diat	$NPCu_{diat}$	input from normalised quota control
**AUXILIARIES**		
pHminop_diat	$pHminop_{diat}$	pHopt_diat-NPCu_diat^NCuH_diat*(pHopt_diat-pHmin_diat)
rpHmin_diat	$rpHmin_{diat}$	(pH>pHminop_diat)*(pH<=pHopt_diat)*(pH-pHminop_diat)/(pHopt_diat-pHminop_diat)+(pH>pHopt_diat)
cpHmin_diat	$cpHmin_{diat}$	(1+pHK_diat^pHH_diat)*(rpHmin_diat^pHH_diat)/(rpHmin_diat^pHH_diat+pHK_diat^pHH_diat)
pHmaxop_diat	$pHmaxop_{diat}$	pHopt_diat+NPCu_diat^NCuH_diat*(pHmax_diat-pHopt_diat)
rpHmax_diat	$rpHmax_{diat}$	(pH<pHmaxop_diat)*(pH>=pHopt_diat)*(pHmaxop_diat-pH)/(pHmaxop_diat-pHopt_diat)+(pH<pHopt_diat)
cpHmax_diat	$cpHmax_{diat}$	(1+pHK_diat^pHH_diat)*(rpHmax_diat^pHH_diat)/(rpHmax_diat^pHH_diat+pHK_diat^pHH_diat)
**OUTPUT**		
pHreg_diat	$pHreg_{diat}$	cpHmax_diat*cpHmin_diat

FORMAL EQUATIONS: Growth Interaction

$$pHminop_{diat} = pHopt_{diat} - NPCU_{diat}{}^{NCuH_{diat}} \times (pHopt_{diat} - pHmin_{diat}$$

$$pHmaxop_{diat} = pHopt_{diat} + NPCU_{diat}{}^{NCuH_{diat}} \times (pHmax_{diat} - pHopt_{diat}$$

$$rpHmin_{diat} = (pH > pHminop_{diat}) \times (pH \leq pHopt_{diat}) \times \frac{pH - pHminop_{diat}}{pHopt_{diat} - pHminop_{diat}} + (pH > pHopt_{diat})$$

$$rpHmax_{diat} = (pH < pHmaxop_{diat}) \times (pH \geq pHopt_{diat}) \times \frac{pHmaxop_{diat} - pH}{pHmaxop_{diat} - pHopt_{diat}} + (pH < pHopt_{diat})$$

$$cpHmin_{diat} = (1 + pHK_{diat}^{pHH_{diat}}) \times \frac{rpHmin_{diat}^{pHH_{diat}}}{rpHmin_{diat}^{pHH_{diat}} + pHK_{diat}^{pHH_{diat}}}$$

$$cpHmax_{diat} = (1 + pHK_{diat}^{pHH_{diat}}) \times \frac{rpHmax_{diat}^{pHH_{diat}}}{rpHmax_{diat}^{pHH_{diat}} + pHK_{diat}^{pHH_{diat}}}$$

$$pHreg_{diat} = cpHmax_{diat} \times cpHmin_{diat}$$

Table S1e Descriptions of parameters for death interactions

PARAMETER	FORMAL NAME	UNIT	DESCRIPTION
**INPUTS**			
pH	Ph	Ph	pH of medium
NPCu_diat	$NPCu_{diat}$	DL	nutrient status from normalised quota equation (0 = poor; 1 is optimal)
**AUXILIARY**			
DR_diat	DR_{diat}	d^{-1}	Death rate

Table S1f Definitions of parameters for death interactions

PARAMETER	FORMAL NAME	DEFINITION
**INPUTS**		
pH	pH	input from DIC chemistry
NPCu_diat	$NPCU_{diat}$	input from normalised quota control
**AUXILIARY**		
DR_diat	DR_{diat}	DRo_diat+(pH<pHopt_diat)*(1-LDD_Diat*NPCu_diat)^lNCu_diat*(ABS(pH-pHopt_diat)*ls_diat)^curv_diat+(pH>pHopt_diat)*(1-HDD_Diat*NPCu_diat)^hNCu_diat*(ABS(pH-pHopt_diat)*hs_diat)^curv_diat

FORMAL EQUATION: Death Interaction

$$DR_{diat} = DRo_{diat} + (pH < pHopt_{diat}) \times (1 - LDD_{diat} \times NPCU_{diat})^{INCu_{diat}} \times (ABS(pH - pHopt_{diat}) \times Is_{diat})^{curve_{diat}} + (pH > pHopt_{diat}) \times (1 - HDD_{diat} \times NPCU_{diat})^{hNCu_{diat}} \times (ABS(pH - pHopt_{diat}) \times hs_{diat})^{curv_{diat}}$$

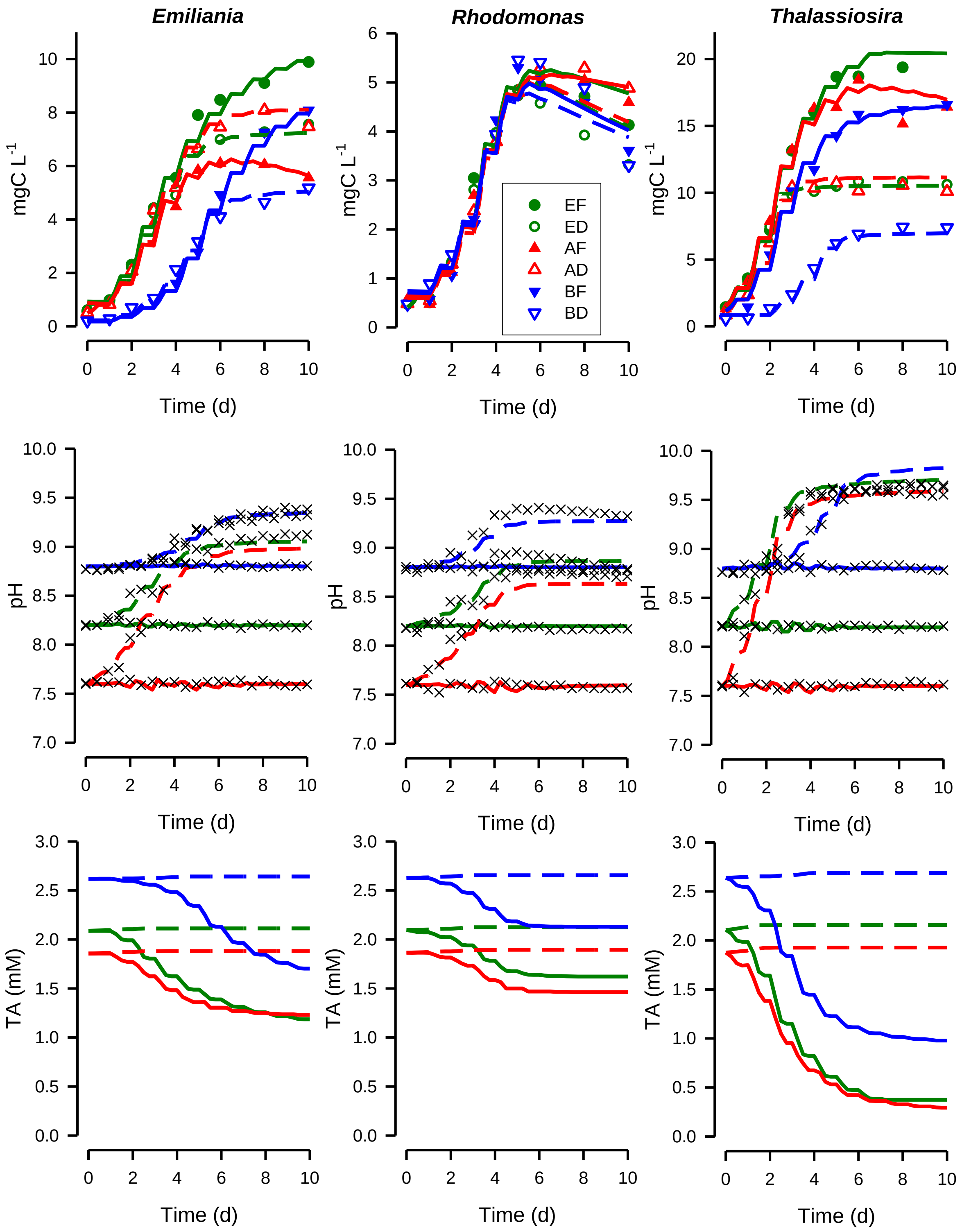

Figure S1. As for figure 1, but also showing changes in pH (with selected experimental data as symbols) and total alkalinity (TA). Conditions of pH were extant fixed at pH 8.2 (EF), extant drifting from pH 8.2 (ED), acidic fixed at pH 7.6 (AF), acidic drifting from pH 7.6 (AD), basic fixed at pH 8.8 (BF) or basic drifting from pH 8.8 (BD). All lines (continuous for solid symbols, dashed for open symbols for the appropriate colour) are model outputs. See figures S3-5 for details on the computed carbonate chemistry.

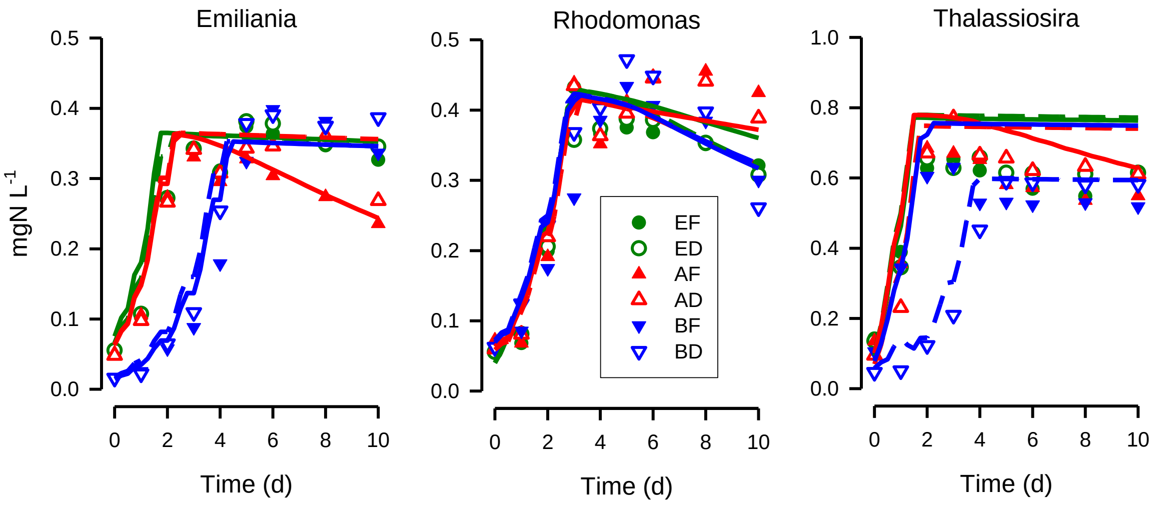

Figure S2. Experimental data and model outputs for algal cellular-N. Experimental data (symbols) are shown for the prymnesiophyte *Emiliania huxleyi*, the cryptophyte *Rhodomonas* sp. and the diatom *Thalassiosira weisflogii* grown under conditions of pH that were extant fixed at pH 8.2 (EF), extant drifting from pH 8.2 (ED), acidic fixed at pH 7.6 (AF), acidic drifting from pH 7.6 (AD), basic fixed at pH 8.8 (BF) or basic drifting from pH 8.8 (BD). Lines are model fits to the data. Experimental data are averages from duplicate experiments, with the range of those values typically within the symbol size. The companion data for algal cellular-C are shown in figures 1 and S1.

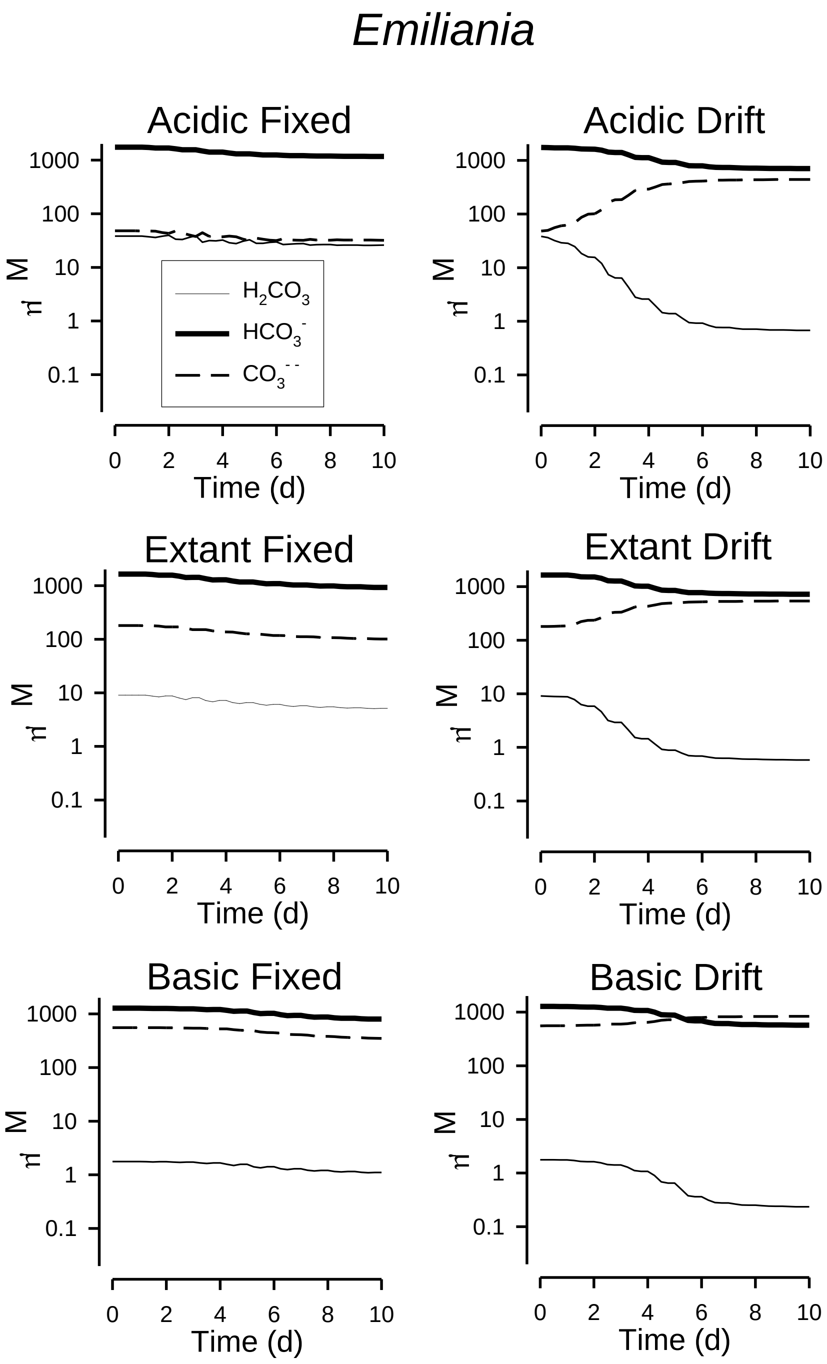

Figure S3. Changes in carbonate chemistry for prymnesiophyte *Emiliania* during growth under conditions of pH that were acidic fixed at pH 7.6 (AF), acidic drifting from pH 7.6 (AD), extant fixed at pH 8.2 (EF), extant drifting from pH 8.2 (ED), basic fixed at pH 8.8 (BF) or basic drifting from pH 8.8 (BD). H_2CO_3 and HCO_3^- are substrates for photosynthesis (see [49]). These values were computed by the model (see model description for comment on carbonate chemistry computation). See also figure S1.

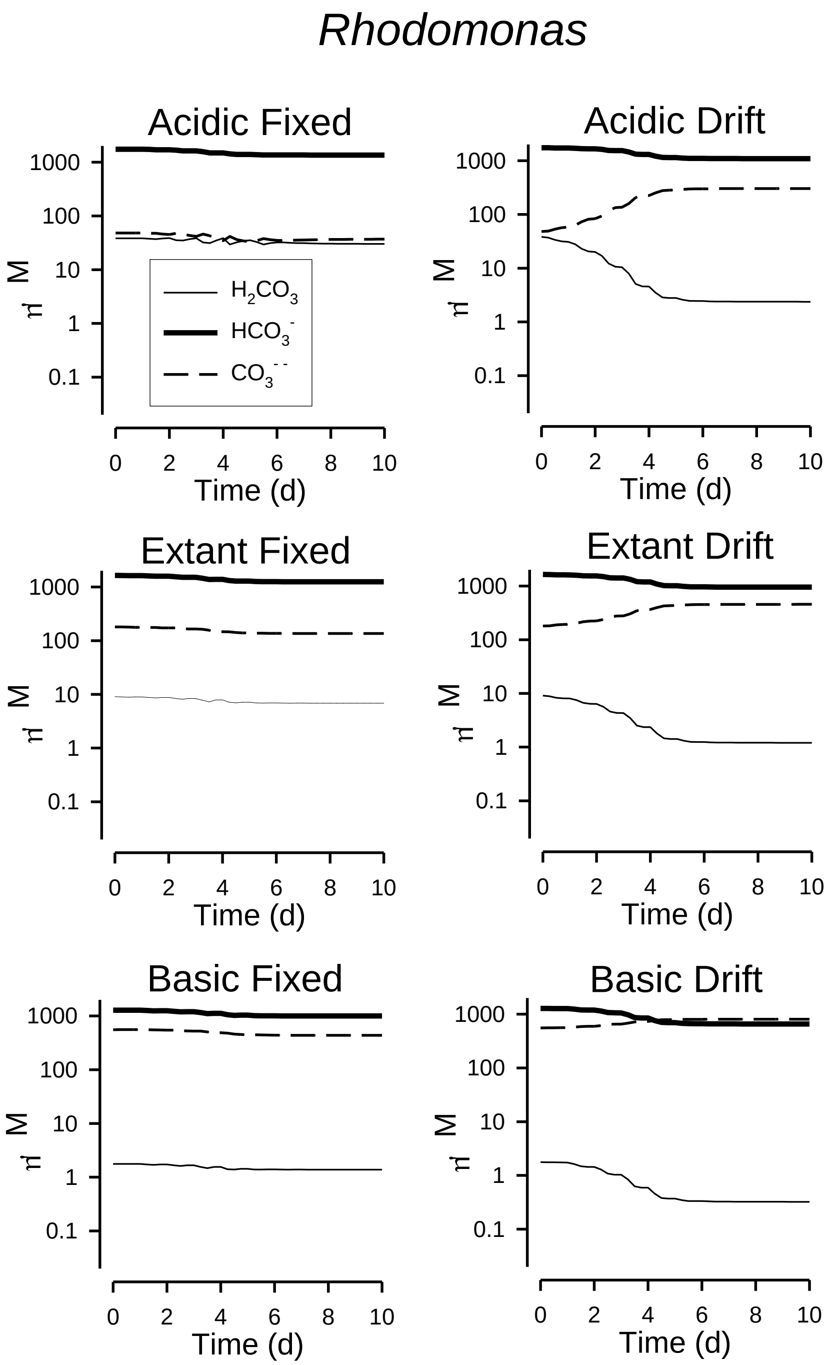

Figure S4. Changes in carbonate chemistry for the cryptophyte *Rhodomonas* during growth under conditions of pH that were acidic fixed at pH 7.6 (AF), acidic drifting from pH 7.6 (AD), extant fixed at pH 8.2 (EF), extant drifting from pH 8.2 (ED), basic fixed at pH 8.8 (BF) or basic drifting from pH 8.8 (BD). H_2CO_3 and HCO_3^- are substrates for photosynthesis (see [49]). These values were computed by the model (see model description for comment on carbonate chemistry computation). See also figure S1.

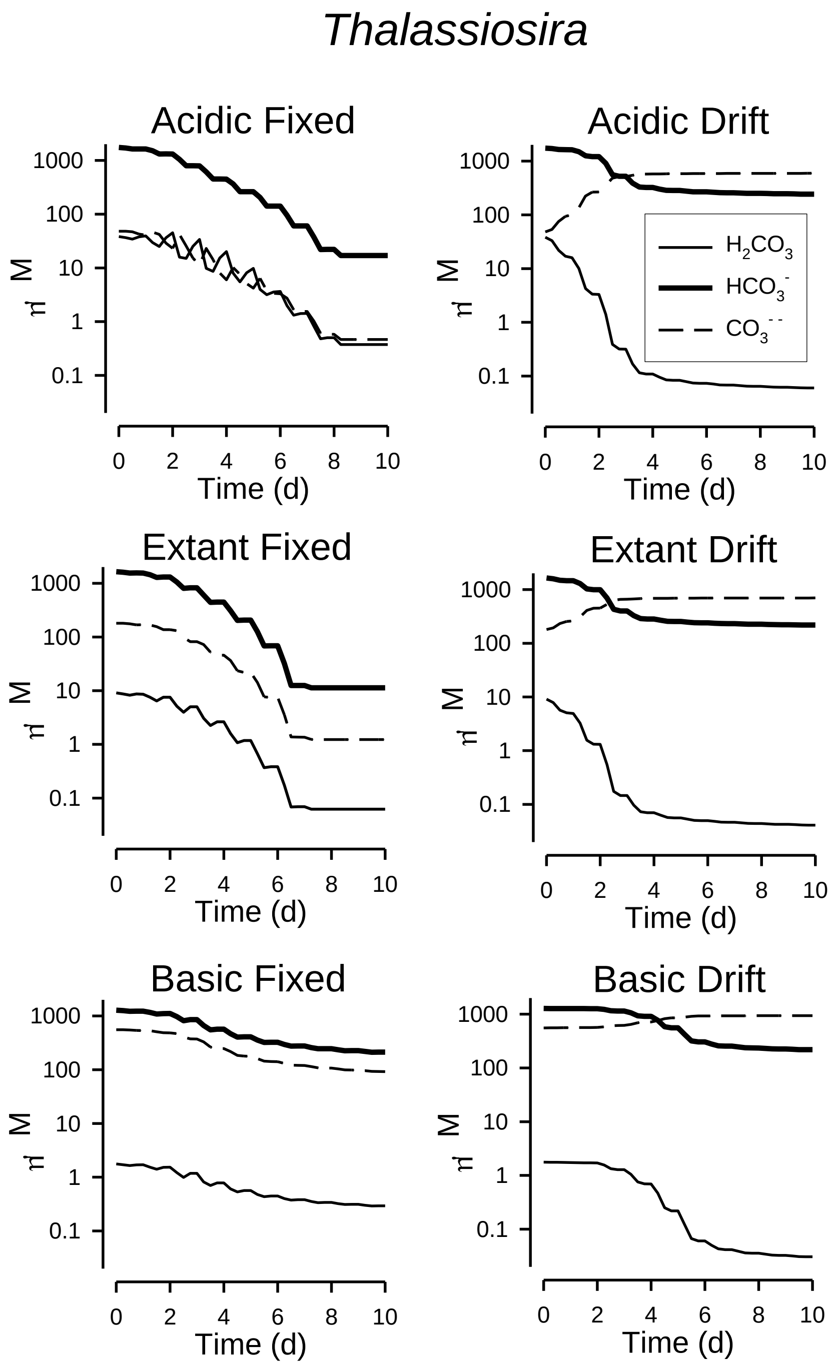

Figure S5. Changes in carbonate chemistry for the diatom *Thalassiosira* during growth under conditions of pH that were acidic fixed at pH 7.6 (AF), acidic drifting from pH 7.6 (AD), extant fixed at pH 8.2 (EF), extant drifting from pH 8.2 (ED), basic fixed at pH 8.8 (BF) or basic drifting from pH 8.8 (BD). H_2CO_3 and HCO_3^- are substrates for photosynthesis (see [49]). These values were computed by the model (see model description for comment on carbonate chemistry computation). See also figure S1.

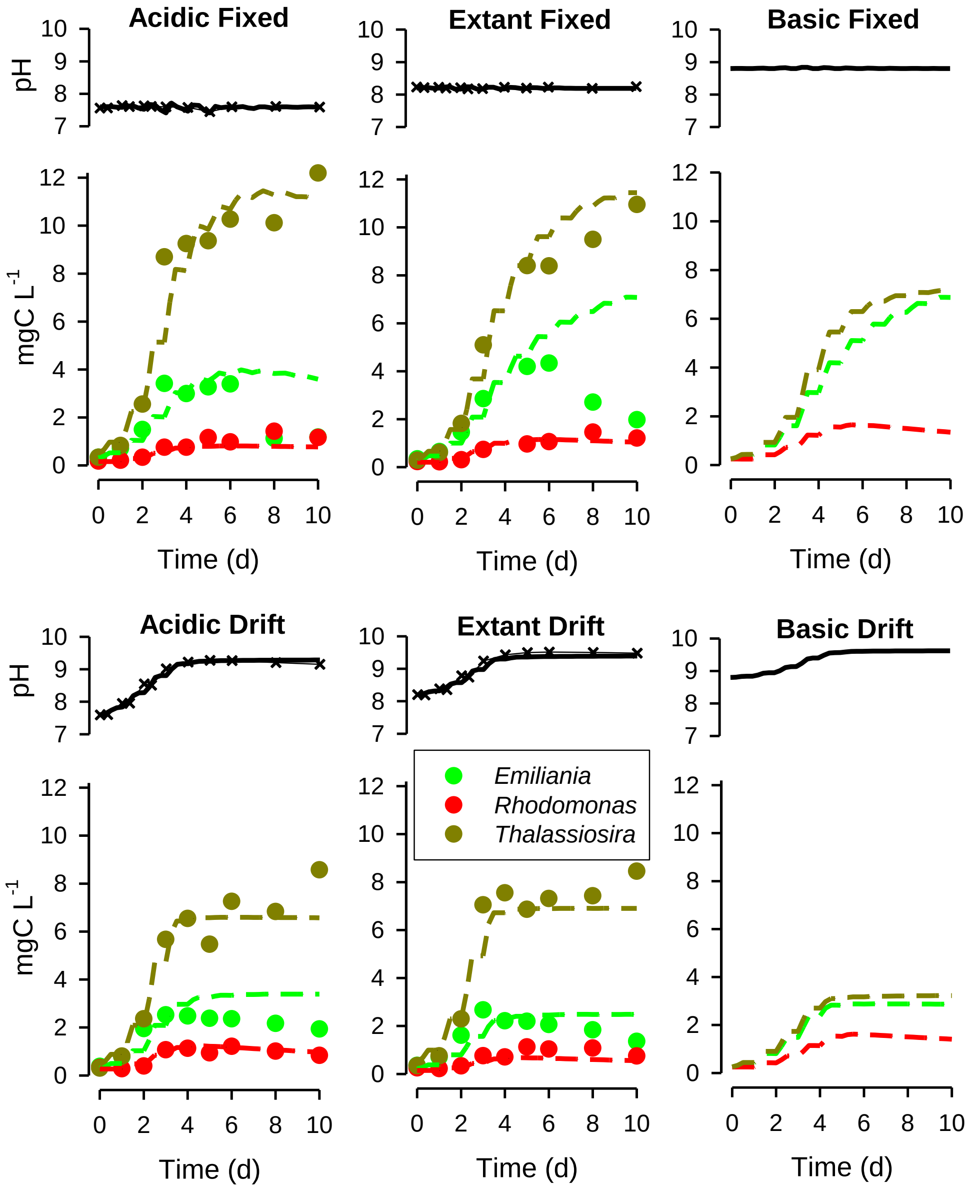

Figure S6. Experimental data (symbols) for the prymnesiophyte *Emiliania huxleyi*, cryptophyte *Rhodomonas sp.* and diatom *Thalassiosira weisflogii* grown together under conditions of pH that were acidic fixed at pH 7.6 (AF), or acidic drifting from pH 7.6 (AD), extant fixed at pH 8.2 (EF) or extant drifting from pH 8.2 (ED). Lines indicate model output; model configurations were those generated from fits to data in figures 1, S1, & S2. Differences between data and model output for *Emiliania huxleyi* likely indicate some form of allelopathic interaction between species that were unaccounted for in the model. Also, shown is simulated growth in mixed cultures for conditions of basic fixed at pH 8.8) and basic drift from pH 8.8; experiments were not conducted under these conditions. Note the value for *Emiliania* in the acidic fixed system at d10 is obscured by the symbol for the cryptophyte.

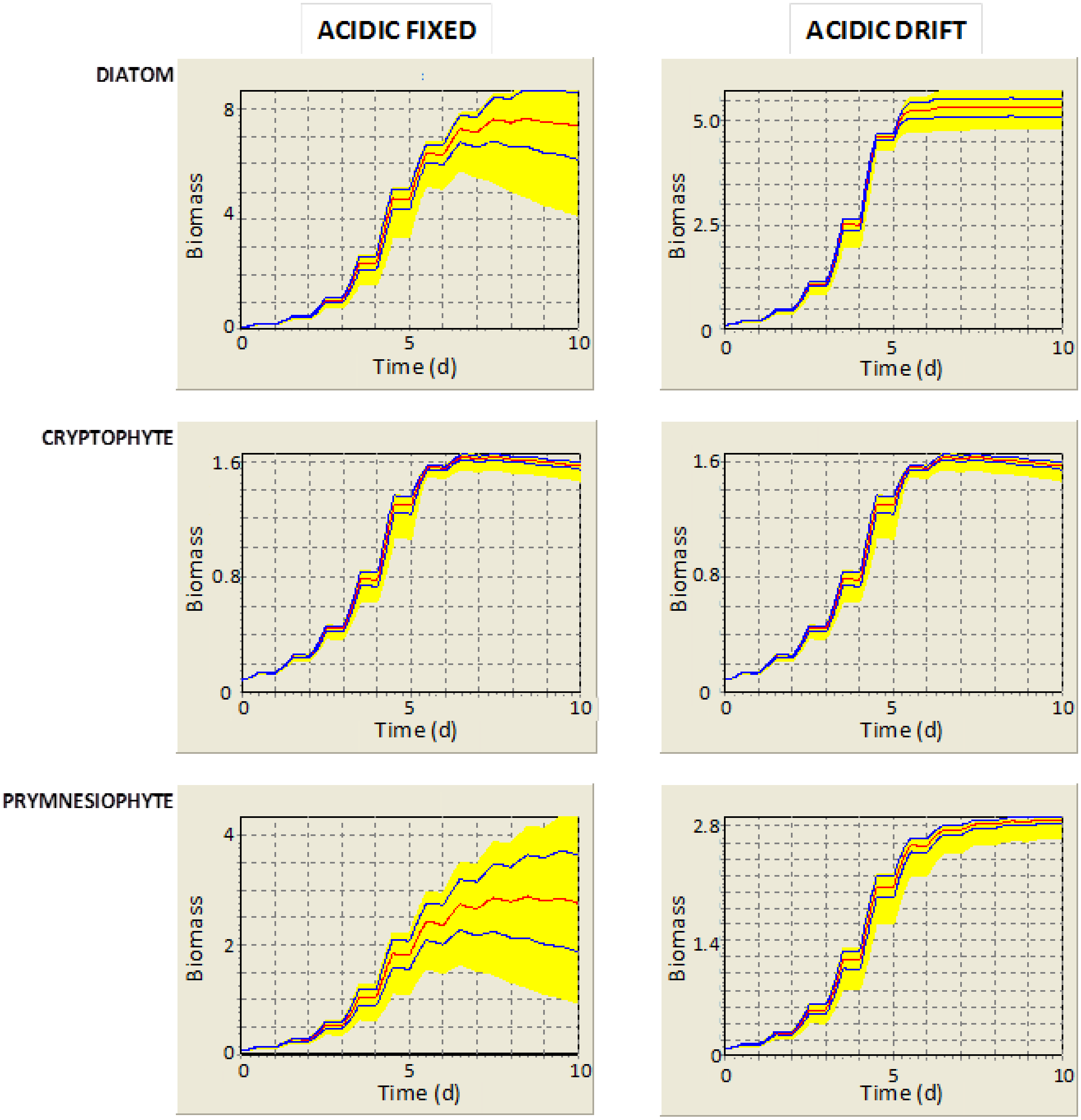

Figure S7. Dynamic sensitivity analysis of growth of mixed species communities commencing with fixed (equal) proportions of start biomass, but with variable "acidic" pH start points, centred at pH 7.6 with standard deviation of pH 0.2 (see model description). Each plot shows, for the indicated phytoplankton type and in a regime with either fixed or drifting pH, the average (red), +/- standard deviation (blue), with the yellow field showing the 95% limits. The relative extent of the 95% confidence limits is the key issue in these plots. Biomass has units of mgC m^{-3}. Systems were ultimately N-limiting.

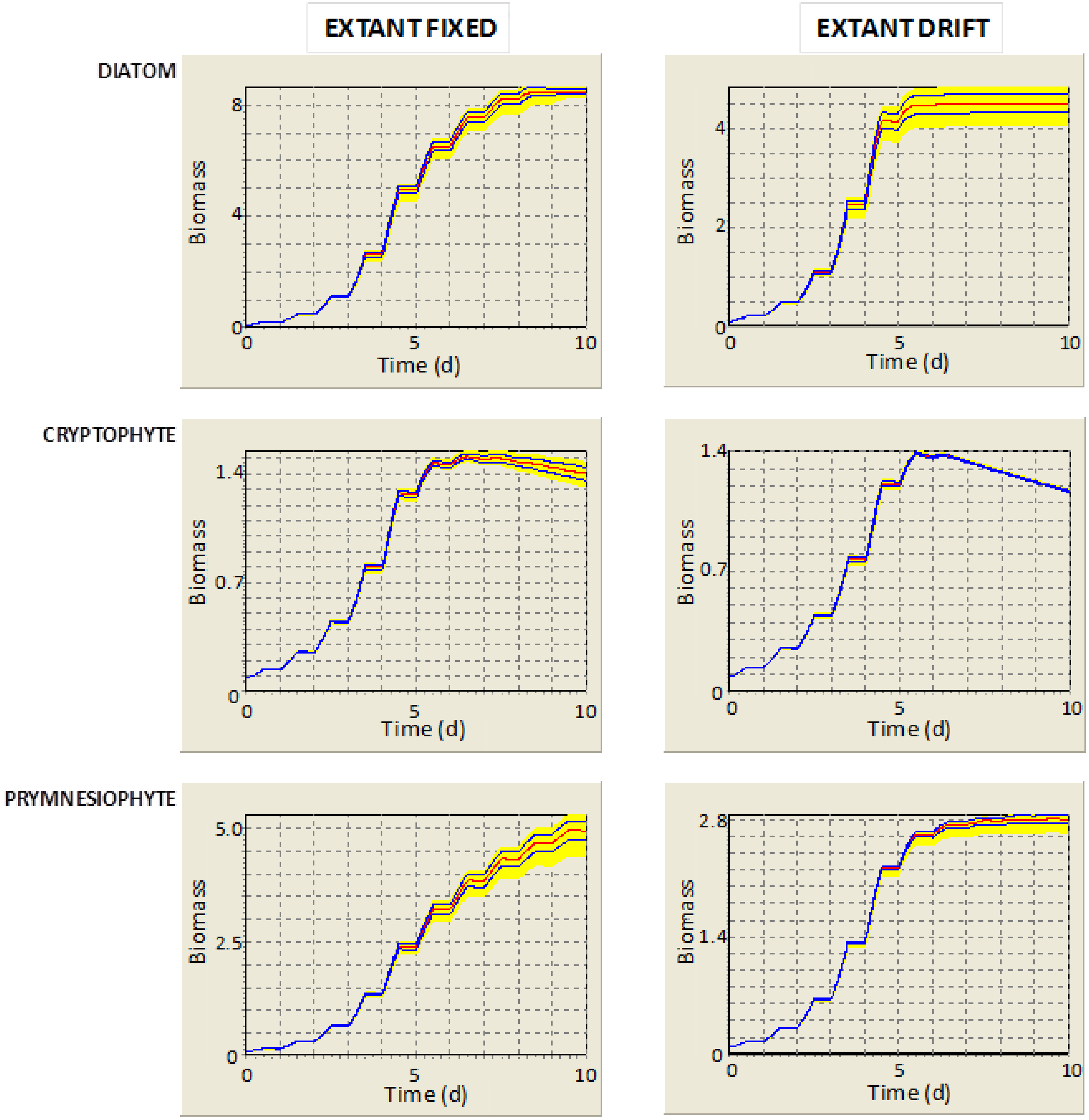

Figure S8. Dynamic sensitivity analysis of growth of mixed species communities commencing with fixed (equal) proportions of start biomass, but with variable "extant" pH start points. The initial pH was centred at pH 8.2 with standard deviation of pH 0.1 (see model description). Each plot shows, for the indicated phytoplankton type and in a regime with either fixed or drifting pH, the average (red), +/- standard deviation (blue), with the yellow field showing the 95% limits. The relative extent of the 95% confidence limits is the key issue in these plots. Biomass has units of mgC m^{-3}. Systems were ultimately N-limiting.

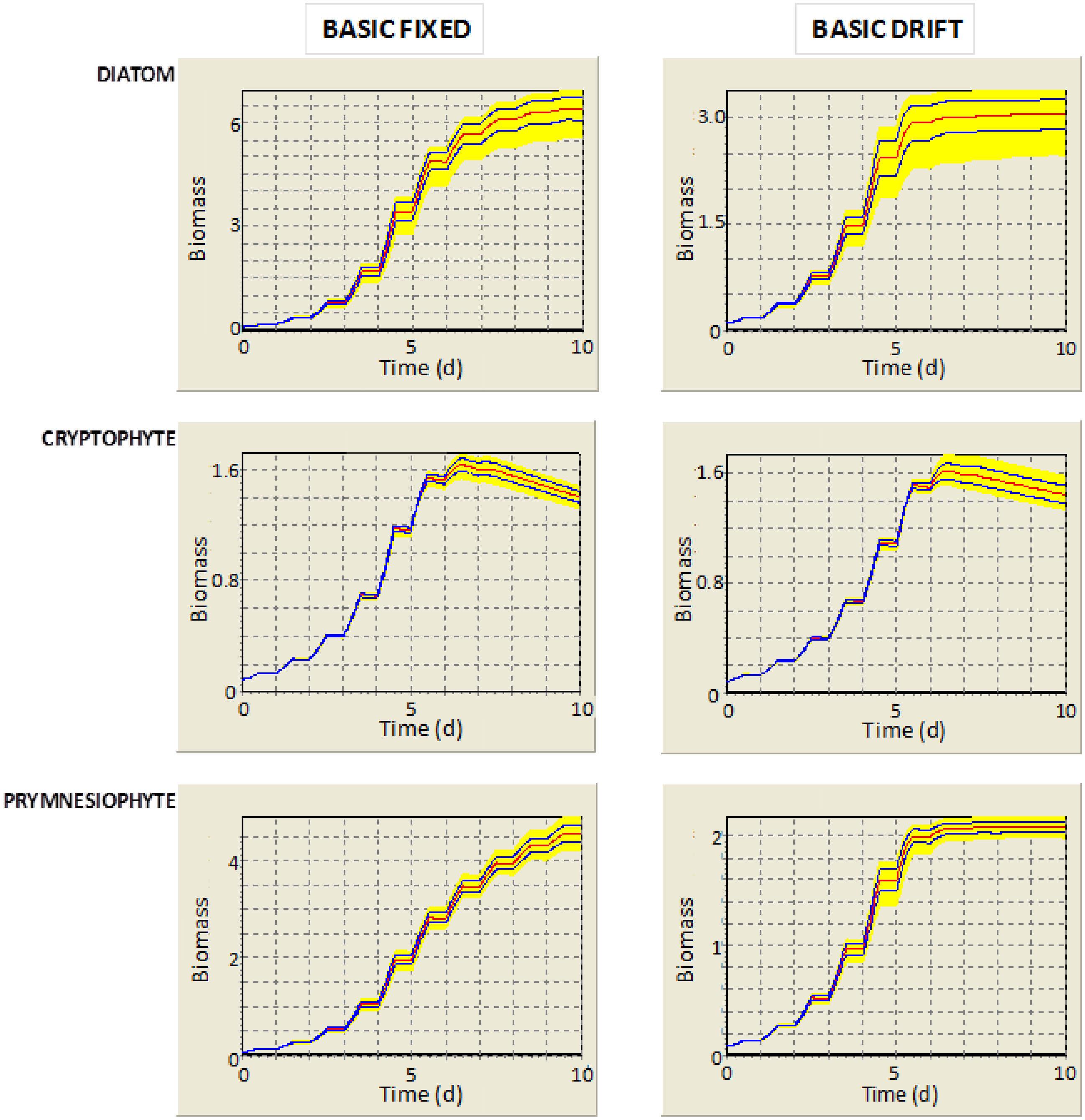

Figure S9. Dynamic sensitivity analysis of growth of mixed species communities commencing with fixed (equal) proportions of start biomass, but with variable "basic" pH start points, centred at pH 8.8 with standard deviation of pH 0.05 (see model description). Each plot shows, for the indicated phytoplankton type and in a regime with either fixed or drifting pH, the average (red), +/- standard deviation (blue), with the yellow field showing the 95% limits. The relative extent of the 95% confidence limits is the key issue in these plots. Biomass has units of mgC m^{-3}. Systems were ultimately N-limiting.

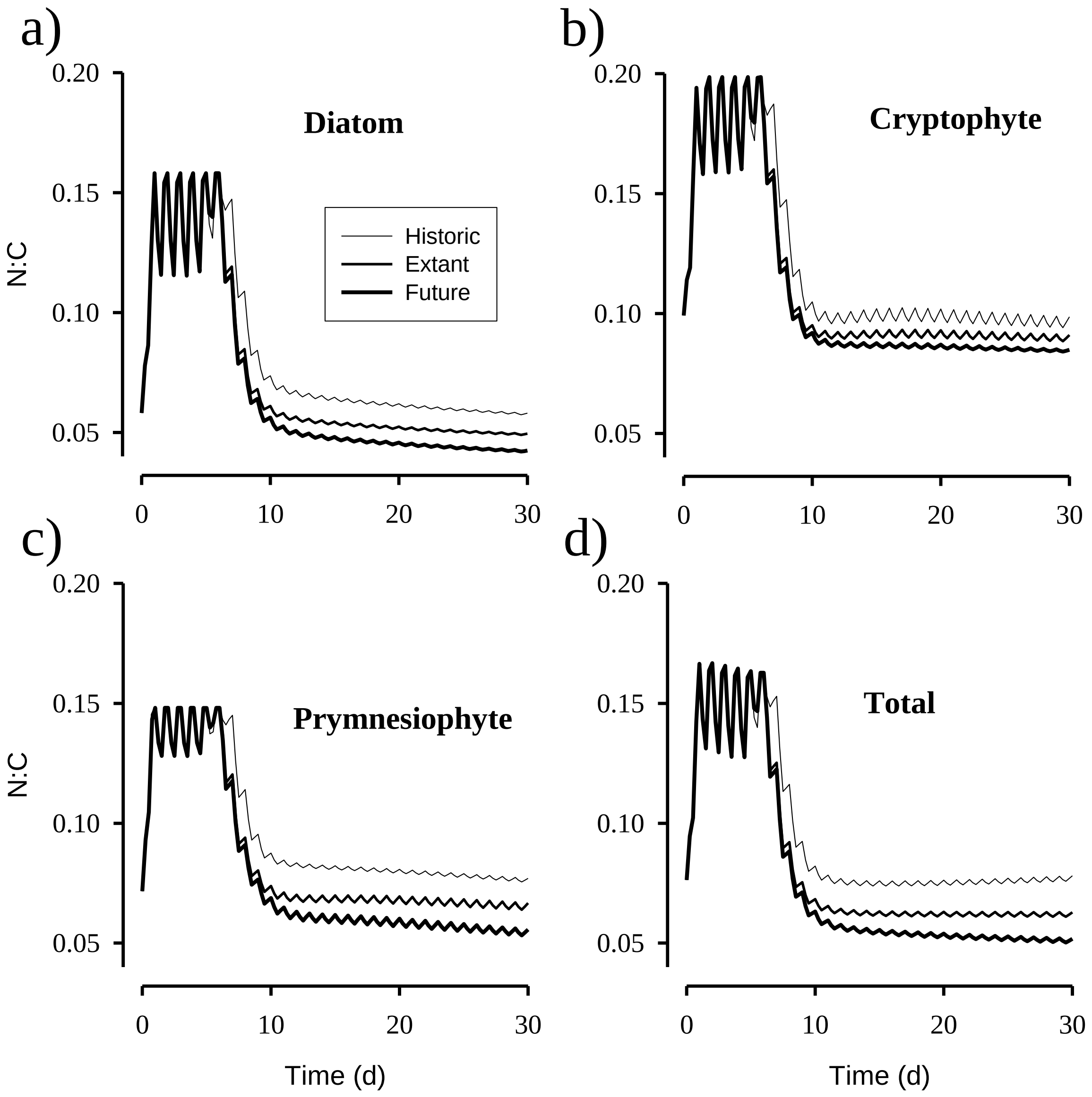

Figure S10. Mass N:C ratios for the simulations within the medium nutrient regime shown in figure 2. Panels (a) to (c) show N:C under historic, extant and future $p\mathrm{CO_2}$ scenarios for the different phytoplankton types indicated. Panel (d) shows the total phytoplankton N:C, which is a function of the individual N:C values and the biomass of the different phytoplankton types (as shown in figure 2, medium nutrient regime series). Oscillations in the values of N:C align with diel fluctuations in physiology; N:C increases during darkness and decreases with photosynthesis during the light. The N:C quality of phytoplankton as zooplankton food would depend on the degree of prey selectivity, and the presence of other factors (such as the presence of noxious secondary metabolites). In consequence, effective prey quality may not relate linearly to elemental stoichiometry [27,28].
